# Full-speed domain position sensorless control strategy for PMSM based on improved super-twisting sliding-mode observer and smooth transition optimization

**DOI:** 10.1038/s41598-026-35994-5

**Published:** 2026-01-19

**Authors:** Xia Zhang, Pengwei Li, Bo Wang, Minghao Lv, Liangtong Shi

**Affiliations:** School of Electrical and Information Engineering, Beihua University, Jilin, 132021 China

**Keywords:** PMSMs, Full speed range position sensorless control, Variable-gain linear super-twisting sliding-mode observer (VGLSTSMO), High-frequency injection (HFI), Sine-weighted switching function, Engineering, Physics

## Abstract

In the sensorless control of permanent magnet synchronous motors (PMSMs), achieving smooth transitions from low to medium–high speeds remains a significant challenge. Aiming at the problem that PMSMs cannot be smoothly transitioned to medium–high speed range, this paper proposes a full-speed range control algorithm based on the fusion of pulsating high-frequency injection (HFI) with square waves and an improved super-twisting sliding-mode observer (STSMO). The proposed method enables smooth and stable operation over the full speed range, with notable improvements observed during transitional phases. In the low-speed range, high-frequency current signal is obtained by injecting a high-frequency (HF) square-wave signal, and the signal is processed to obtain rotor position and speed information. For medium–high speeds, a variable-gain linear STSMO (VGLSTSMO) combined with an adaptive back electromotive force (back-EMF) model is employed to improve robustness. Furthermore, this paper designs a Sine-weighted switching function to facilitate a smooth transition of motors from low to medium–high speeds domains. The effectiveness and superiority of the proposed methods are validated through simulations. The rotor position estimation errors of the improved STSMO method are approximately 0.2 rad, and Sine-weighted switching method enables motors to switch smoothly from the low-speed range to the medium- high-speed ranges.

## Introduction

Permanent magnet synchronous motors (PMSMs) are widely utilized in advanced applications such as aerospace, marine propulsion, and new energy vehicles due to their compact size, high power density, and excellent speed regulation capabilities^1^. Conventional vector control systems typically rely on mechanical sensors to acquire real-time rotor position and speed information. However, the mechanical sensors and associated signal-processing modules not only increases system cost and hardware complexity but also degrades operational reliability under harsh environmental conditions^2^.Therefore, the advancement of sensorless control technology is of great significance for improving system reliability, reducing costs, and facilitating energy conservation and emission reduction^3^.Against this backdrop, field-oriented control (FOC) has attracted considerable attention as an advanced strategy capable of effectively reducing the control complexity of PMSMs systems.

Currently, Sensorless control methods for PMSMs can be categorized into two main types: the back electromotive force (back-EMF) method, which is suitable for medium–high-speed ranges, and the high-frequency signal injection method, which is applicable to low-speed ranges and relies on the motor’s salient pole characteristics^4^. At low speeds, in addition to the high-frequency signal injection method^5^, I/F control is also a widely adopted control strategy. Reference^6^ proposed a strategy combining improved I/F control with the effective flux linkage method, addressing the issue of low-speed observation accuracy. However, I/F control is inherently an open-loop scheme that relies on high-current startup, leading to problems such as low current utilization efficiency, susceptibility to loss of synchronization, and slow startup. In order to further improve the dynamic performance in the low-speed section, current research commonly utilizes high-frequency signal injection methods to achieve speed closed-loop control. The high-frequency signal injection method enables high-precision position observation in the low-speed range by injecting specific forms of high-frequency voltage or current excitation (such as rotating or pulsating sine waves, or square waves) into the motor^7,8^, and subsequently extracting rotor position and speed information based on the response signals. Reference^9^ proposed a full-speed-range sensorless composite control strategy for PMSMs with rotating high-frequency injection and Improving sliding mode observer. However, the low-speed method still relies on low-pass filters to extract high-frequency response signals, inevitably introducing phase lag and amplitude attenuation. In contrast, the square-wave injection method replaces conventional filtering with differential processing, effectively resolving these issues and improving position observation accuracy and response speed^10^^.^

Observation methods based on back EMF mainly include the sliding mode observer (SMO)^11^, extended state observer (ESO)^12^, extended Kalman filter (EKF) algorithm^13^, and flux linkage observer^14^. Reference^15^ proposed a sensorless control scheme based on the integration of model predictive control (MPC) and EKF. The EKF method has strong anti-interference ability, but the calculation is very complicated. The flux linkage observer is affected by the dead zone of the inverter. The SMO method has advantages such as strong robustness, fast dynamic response, and simple structure^16^. However, SMO inherently suffers from chattering issues and typically requires a low-pass filter (LPF) to extract rotor position information from back EMF, which may introduce phase delay and deteriorate the estimation precision. This method also weakens the advantage of variable structure control. Consequently, higher-order SMO have gained increasing application. The super-twisting sliding-mode observer (STSMO), as a representative second-order sliding mode algorithm, is extensively employed in sensorless control of PMSMs. It effectively suppresses chattering while enhancing the accuracy of speed and position estimation. Reference^17^ designs a second-order SMO by adopting a super-twisting algorithm that effectively suppresses the chattering phenomenon. However, this approach utilizes a fixed sliding mode gain, which limits the estimation accuracy of back EMF, resulting in steady-state errors in speed and position estimation^18^.

To achieve a smooth transition of the motor from low to medium–high speeds, commonly used switching schemes include the hysteresis switching and the weighted switching method^19^. The hysteresis switching method is relatively simple and can effectively suppress frequent switching near the critical point in the transition region, but it may induce position and speed fluctuations. The weighted switching method generally applies weighting to the rotational speed or position, which can reduce fluctuations in both variables within the transition zone; however, it may still cause abrupt changes in torque and rotational speed. Reference^20^ proposed a full-speed-range sensorless composite control strategy for PMSMs with high-frequency square-wave injection and HSMO to mitigate phase delay and chattering issues in traditional SMO. It employs a linear weighted switching strategy in the transition region; however, its weighting coefficients do not sufficiently account for dynamic variations in observer error under different operating conditions. Table 1 provides a comparative summary of the main contributions and limitations of recent relevant studies.

**Table 1:** Sensorless control schemes for PMSM.

References	Main Contributions	Limitations
Yao et al., Control Engineering Practice (2024)^3^	Proposed a full-speed domain sensorless strategy using I/F startup, Luenberger observer, and improved PLL	Strongly depends on PLL tuning; system complexity is relatively high and transitions may introduce phase lag
Gao et al., Scientific Reports (2024)^21^	proposed a control strategy combining pulsed high-frequency square-wave injection with a high-order SMO (HSMO)	The HSMO maintains fixed gain coefficients, limiting its disturbance rejection capability and system robustness
Shi et al., Processes (2024)^22^	Proposed an improved adaptive super-twisting sliding mode observer (ASTSMO) with an extended state observer (ESO)	Only in the medium–high speed region; full-speed domain control was not addressed
Our work	Proposes a full-speed domain control algorithm combining square-wave HFI, variable-gain linear STSMO (VGLSTSMO), and a sinusoidal-weighted switching function	This scheme deserves further study

The main contributions of this paper can be summarized as follows:(1) An improved STSMO is proposed. By designing variable gain coefficients to effectively suppress high-frequency chattering and introducing an adaptive back-EMF mechanism, The variable-gain linear STSMO (VGLSTSMO) significantly reduces state observation errors in the medium–high-speed range, thereby enhancing the system’s robustness across the full speed range.(2) Designing a sine-weighted switching function to facilitate smooth transitions between the two control methods within the transition interval, thereby enhancing speed switching smoothness and system dynamic performance.(3) proposed a full-speed-range sensorless composite control strategy for PMSM with high-frequency square-wave injection and VGLSTSMO. By dynamically allocating the control output ratio across different speed ranges through a sine-switching weighting function, it achieves smooth transitions and stable operation. (4) MATLAB/Simulink simulation results validate the feasibility and superiority of the proposed strategy, providing an effective solution for full-speed-range sensorless control of PMSMs.

The remaining portion of this paper is organized as follows: In Section “Sensorless control methods for PMSMs”, the mathematical models of PMSM, HFI with square waves, and STSMO are derived. Section “Improved STSMO method” presents the mathematical formulations of the VGLSTSMO and the design principles of the adaptive back-EMF. Section “Smooth switching strategy for full-speed-range sensorless composite control” elaborates on the proposed composite control strategy and switching methods across the full-speed range. Section “Simulation verification and results analysis” provides the simulation results and corresponding performance analysis. Finally, Section “Conclusions” summarizes the paper.

## Sensorless control methods for PMSMs

### Mathematical model of PMSMs

The voltage equations of PMSMs in the two-phase rotating coordinate system (dq-axis) can be written as follows:

1 $$\left[ {\begin{array}{*{20}c} {u_{d} } \\ {u_{q} } \\ \end{array} } \right] = R\left[ {\begin{array}{*{20}c} {i_{d} } \\ {i_{q} } \\ \end{array} } \right] + \left[ {\begin{array}{*{20}c} {L_{d} } & 0 \\ 0 & {L_{q} } \\ \end{array} } \right]\frac{d}{dt}\left[ {\begin{array}{*{20}c} {i_{d} } \\ {i_{q} } \\ \end{array} } \right] + \omega_{e} \left[ {\begin{array}{*{20}c} 0 & { - L_{q} } \\ {L_{d} } & 0 \\ \end{array} } \right]\left[ {\begin{array}{*{20}c} {i_{d} } \\ {i_{q} } \\ \end{array} } \right] + \left[ {\begin{array}{*{20}c} 0 \\ {\omega_{e} \psi_{f} } \\ \end{array} } \right]$$

where ${u}_{d} ,{u}_{q}$ are the dq-axis stator voltages and ${i}_{d} ,{i}_{q}$ are the dq-axis current components. ${L}_{d}$ and ${L}_{q}$ are the dq-axis inductances of the motor, and ${\omega }_{e}$ and ${\uppsi }_{\mathrm{f}}$ represent the rotor electrical angular velocity and flux linkage, respectively.

The voltage equations of PMSMs in the two-phase stationary coordinate system ($\alpha \beta$-axis) can be written as follows:

2 $$\left[ {\begin{array}{*{20}c} {u_{\alpha } } \\ {u_{\beta } } \\ \end{array} } \right] = \left[ {\begin{array}{*{20}c} {R + \frac{d}{dt}L_{d} } & {\omega_{e} (L_{d} - L_{q} )} \\ { - \omega_{e} \psi_{f} (L_{d} - L_{q} )} & {R + \frac{d}{dt}L_{d} } \\ \end{array} } \right]\left[ {\begin{array}{*{20}c} {i_{\alpha } } \\ {i_{\beta } } \\ \end{array} } \right] + \left[ {\begin{array}{*{20}c} {e_{\alpha } } \\ {e_{\beta } } \\ \end{array} } \right]$$

3 $$\left[ {\begin{array}{*{20}c} {e_{\alpha } } \\ {e_{\beta } } \\ \end{array} } \right] = \left[ {\omega_{e} \psi_{f} + (L_{d} - L_{q} )(\omega_{e} i_{d} - \frac{d}{dt}i_{q} )} \right]\left[ {\begin{array}{*{20}c} { - \sin \theta } \\ {\cos \theta } \\ \end{array} } \right]$$

where $u_{\alpha }$ , $u_{\beta }$ are the $\alpha \beta$-axis stator voltages and $i_{\alpha }$ and $i_{\beta }$ are the $\alpha \beta$-axis corresponding current components, and $e_{\alpha }$, $e_{\beta }$ represents the extended back-EMF in the $\alpha \beta$-axis.

Rewrite Eq. (2) in state-space form, with current as the state variable, the following expression is obtained:

4 $$\frac{d}{dt}\left[ {\begin{array}{*{20}c} {\hat{i}_{\alpha } } \\ {\hat{i}_{\beta } } \\ \end{array} } \right] = \left[ {\begin{array}{*{20}c} { - \frac{R}{{L_{d} }}} & { - \frac{{\omega_{e} (L_{d} - L_{q} )}}{{L_{d} }}} \\ {\frac{{\omega_{e} (L_{d} - L_{q} )}}{{L_{d} }}} & { - \frac{R}{{L_{d} }}} \\ \end{array} } \right]\left[ {\begin{array}{*{20}c} {\hat{i}_{\alpha } } \\ {\hat{i}_{\beta } } \\ \end{array} } \right] + \frac{1}{{L_{d} }}\left[ {\begin{array}{*{20}c} {u_{\alpha } } \\ {u_{\beta } } \\ \end{array} } \right] - \frac{1}{{L_{d} }}\left[ {\begin{array}{*{20}c} {e_{\alpha } } \\ {e_{\beta } } \\ \end{array} } \right]$$

The electromagnetic torque equation of PMSMs can be expressed as:

5 $$T_{e} = \frac{3}{2}P_{n} \left[ {i_{q} \psi_{f} + i_{d} i_{q} (L_{d} - L_{q} )} \right]$$

where ${\mathrm{T}}_{\mathrm{e}}$ is the electromagnetic torque, ${\mathrm{P}}_{\mathrm{n}}$ is number of pole pairs.

The mechanical motion equation of PMSMs can be expressed as:

6 $$J\frac{{d\omega_{m} }}{dt} = T_{e} - T_{L} - B\omega_{m}$$

where ${\upomega }_{\mathrm{m}}$ is the mechanical angular velocity, J is the moment of inertia, B is the damping coefficient, and ${T}_{L}$ is the load torque.

### Pulsating high-frequency square-wave voltage injection method

**Fig. 1:**
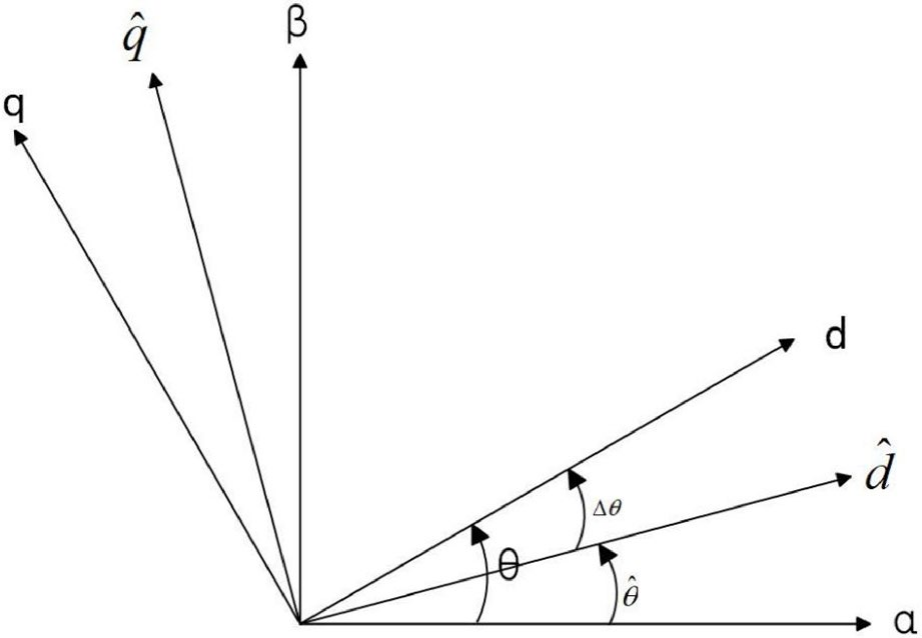
Angular Diagram of the Relationship between Coordinate Axis.

In the full-speed-range sensorless control strategy, the pulsating high-frequency square-wave voltage injection method is used to estimate rotor position and speed in the low-speed range. First, the relationship between the estimated rotor synchronous rotating coordinate system ($\hat{d}\hat{q}$) and the actual rotor synchronous rotating coordinate system (dq) is established, as shown in Fig. 1.

In Fig. 1, $\hat{\theta }$ represents the estimated rotor position angle, $\uptheta$ represents the actual rotor position angle, and $\Delta\uptheta$ represents the rotor position angle error.

At low speeds, the motor operates at extremely low rotational speeds, making the back-EMF magnitude negligible. Meanwhile, when high-frequency voltage signals are injected into the dq-axis, the inductive reactance of the windings significantly exceeds the resistive reactance, allowing the effect of resistive voltage drops to be neglected. Based on these simplifications, the high-frequency response model of the system can be expressed as:

7 $$\left[ {\begin{array}{*{20}c} {u_{dh} } \\ {u_{qh} } \\ \end{array} } \right] = \left[ {\begin{array}{*{20}c} {L_{dh} } & 0 \\ 0 & {L_{qh} } \\ \end{array} } \right]\frac{d}{dt}\left[ {\begin{array}{*{20}c} {i_{dh} } \\ {i_{qh} } \\ \end{array} } \right]$$

where $u_{dh}$ , $u_{qh}$ are the dq-axis high-frequency voltage components, and $i_{dh}$ , $i_{qh}$ are the corresponding high-frequency current components.

Based on the inverse Park transformation, the high-frequency mathematical model in the coordinate system can be derived as:

8 $$\left[ {\begin{array}{*{20}c} {u_{\alpha h} } \\ {u_{\beta h} } \\ \end{array} } \right] = \left[ {\begin{array}{*{20}c} {\cos \theta } & { - \sin \theta } \\ { - \sin \theta } & {\cos \theta } \\ \end{array} } \right]\left[ {\begin{array}{*{20}c} {u_{dh} } \\ {u_{qh} } \\ \end{array} } \right]$$

where $u_{\alpha h}$ , $u_{\beta h}$ are the $\alpha \beta$-axis high-frequency voltage components.

Substituting Eq. (7) into Eq. (8) and applying the Park transformation results in the following expression:

9 $$\left[ {\begin{array}{*{20}c} {u_{\alpha h} } \\ {u_{\beta h} } \\ \end{array} } \right] = \left[ {\begin{array}{*{20}c} {L - \Delta L\cos 2\theta } & { - \Delta L\sin 2\theta } \\ { - \Delta L\sin 2\theta } & {L + \Delta L\cos 2\theta } \\ \end{array} } \right]\frac{d}{dt}\left[ {\begin{array}{*{20}c} {i_{\alpha h} } \\ {i_{\beta h} } \\ \end{array} } \right]$$

where $i_{\alpha h}$,$i_{\beta h}$ are the $\alpha \beta$-axis high-frequency current components, $L = \frac{{L_{q} + L_{d} }}{2}$ is the average inductance, and $\Delta L = \frac{{L_{q} - L_{d} }}{2}$ is the half-difference inductance.

In sensorless control algorithms, since the actual dq-axis positions cannot be directly obtained, an estimated $\hat{d}\hat{q}$-axis coordinate system be introduced. By transforming the Eqs. (9) into the $\hat{d}\hat{q}$-axis and extracting the high-frequency response components of the current, the following expression can be derived:

10 $$\frac{d}{dt}\left[ {\begin{array}{*{20}c} {i_{dh} } \\ {i_{qh} } \\ \end{array} } \right] = \left[ {\begin{array}{*{20}c} {\frac{L + \Delta L\cos 2\theta }{{L^{2} - \Delta L^{2} }}} & {\frac{\Delta L\sin 2\theta }{{L^{2} - \Delta L^{2} }}} \\ {\frac{\Delta L\sin 2\theta }{{L^{2} - \Delta L^{2} }}} & {\frac{L - \Delta L\cos 2\theta }{{L^{2} - \Delta L^{2} }}} \\ \end{array} } \right]\left[ {\begin{array}{*{20}c} {u_{{\hat{d}h}} } \\ {u_{{\hat{q}h}} } \\ \end{array} } \right]$$

A high-frequency voltage square-wave signal is injected into the d-axis.

11 $$\left[ {\begin{array}{*{20}c} {u_{{\hat{d}h}} } \\ {u_{{\hat{q}h}} } \\ \end{array} } \right] = \left[ {\begin{array}{*{20}c} { \pm U_{in} } \\ 0 \\ \end{array} } \right]$$

Substituting Eq. (11) into Eq. (10) and integrating yields the high-frequency current response containing rotor position information, which can be derived as:

12 $$\left[ {\begin{array}{*{20}c} {i_{{\hat{d}h}} } \\ {i_{{\hat{q}h}} } \\ \end{array} } \right] = \frac{{ \pm U_{in} T_{s} }}{{L^{2} - \Delta L^{2} }}\left[ {\begin{array}{*{20}c} {L + \Delta L\cos 2\theta } \\ {\Delta L\sin 2\theta } \\ \end{array} } \right]$$

$i_{{\hat{d}h}}$ and $i_{{\hat{q}h}}$ contain rotor position information. By decoupling the error information through vector cross-multiplication, which can be obtained:

13 $$f(\Delta \theta ) = k\Delta \theta ,k = \frac{{ \pm U_{in} T_{s} (L_{q} - L_{d} )}}{{L_{q} L_{d} }}$$

In Eq. (13), the error signal $f(\Delta \theta )$ represents the deviation between the estimated and actual rotor angles, exhibiting an approximately linear relationship with their angular difference. The proportional coefficient $k$ is determined by the injected voltage amplitude, motor inductance parameters, and the sampling time. The sampling time is set to ${T}_{s}=10 \, \mu s$ in the simulation. The injected voltage amplitude is ${U}_{in}=80V$.

Equation (13) into a phase-locked loop (PLL) enables the extraction of both rotor position and speed information. The closed-loop transfer function of PLL as:

14 $$G(s) = \frac{{\tilde{\theta }_{e} }}{\theta } = \frac{{k_{p} s + k_{i} }}{{s^{2} + k_{p} s + k_{i} }}$$

where ${k}_{p}$ and ${k}_{i}$ are PI controller parameters, and ${k}_{p}>0$, ${k}_{i}>0$.

### Conventional super-twisting sliding mode observer

STSMO is a second-order sliding mode observer, and its mathematical expression can be expressed as:

15 $$\left\{ {\begin{array}{*{20}c} {\frac{{d\hat{x}_{1} }}{dt} = - k_{1} \sqrt {\left| {x_{1} - \hat{x}_{1} } \right|} \cdot sign(x_{1} - \hat{x}_{1} ) + \hat{x}_{2} + \rho_{1} } \\ {\frac{{d\hat{x}_{2} }}{dt} = - k_{2} \cdot sign(x_{1} - \hat{x}_{1} ) + \rho_{2} } \\ \end{array} } \right.$$

where ${x}_{i}$ is the state variable, $\hat{x}_{i}$ is the state variable estimate, ${\rho }_{i}$ is the disturbance term, ${k}_{1,}{k}_{2}$ are the observer gain parameter, and ${k}_{1,}{k}_{2}>0$.

The super-twisting algorithm introduced in the STSMO method enhances the accuracy and robustness of estimates, particularly for systems with uncertainties and external disturbances. As shown in Eq. (15), the sign function resides within the integral term, effectively performing an integration operation on the sign function to filter and reduce chattering. The sign function is defined as:

16 $$sign(x_{1} - \hat{x}_{1} ) = \left\{ {\begin{array}{*{20}c} { - 1,} \\ {0,} \\ {1,} \\ \end{array} } \right.\begin{array}{*{20}c} {x_{1} - \hat{x}_{1} < 0} \\ {x_{1} - \hat{x}_{1} = 0} \\ {x_{1} - \hat{x}_{1} > 0} \\ \end{array}$$

Let δ is a constant greater than 0 and satisfies the following conditions:

17 $$\delta \ge \frac{{\rho_{1} }}{{\sqrt {\left| {x_{1} } \right|} }}$$

When the disturbance term ${\rho }_{2}=0$, to ensure that the system achieves global stability, the values of the sliding mode variable gain parameter must lie within the following ranges:

18 $$\left\{ {\begin{array}{*{20}c} {k_{1} \ge 2\delta } \\ {k_{2} \ge k_{1} \frac{{5\delta k_{1} + 4\delta^{2} }}{{2(k_{1} - 2\delta )}}} \\ \end{array} } \right.$$

Therefore, incorporating SMO system designed with the super-twisting algorithm ensures that the system state converges to zero within a finite time.

By applying the super-twisting algorithm to the sensorless control of PMSM, the current estimation equation based on STSMO is designed as follows:

19 $$\left\{ {\begin{array}{*{20}c} {\frac{{d\hat{i}_{\alpha } }}{dt} = - \frac{R}{{L_{q} }}\hat{i}_{\alpha } + \frac{1}{{L_{q} }}u_{\alpha } + \frac{1}{{L_{q} }}k_{1} \sqrt {\left| {\tilde{i}_{\alpha } } \right|} \cdot sign(\tilde{i}_{\alpha } ) + \frac{1}{{L_{q} }}\int {k_{2} sign(\tilde{i}_{\alpha } )dt} } \\ {\frac{{d\hat{i}_{\beta } }}{dt} = - \frac{R}{{L_{q} }}\hat{i}_{\beta } + \frac{1}{{L_{q} }}u_{\beta } + \frac{1}{{L_{q} }}k_{1} \sqrt {\left| {\tilde{i}_{\beta } } \right|} \cdot sign(\tilde{i}_{\beta } ) + \frac{1}{{L_{q} }}\int {k_{2} sign(\tilde{i}_{\beta } )dt} } \\ \end{array} } \right.$$

where $\hat{i}_{\alpha } ,\hat{i}_{\beta }$ is the estimated stator current and $\tilde{i} = \hat{i} - i$ is the current estimation error.

According to Eq. (15), the disturbance term can be derived from Eq. (19) as:

20 $$\left\{ {\begin{array}{*{20}c} {\rho_{1} (\hat{i}_{\alpha } ) = - \frac{R}{{L_{q} }}\hat{i}_{\alpha } + \frac{1}{{L_{q} }}u_{\alpha } \le \delta \sqrt {\left| {\hat{i}_{\alpha } } \right|} } \\ {\rho_{1} (\hat{i}_{\beta } ) = - \frac{R}{{L_{q} }}\hat{i}_{\beta } + \frac{1}{{L_{q} }}u_{\beta } \le \delta \sqrt {\left| {\hat{i}_{\beta } } \right|} } \\ \end{array} } \right.$$

When the design parameter $\updelta$ is set sufficiently large, the disturbance term satisfies the stability requirement.

From Eqs. (19) and (4), the error of the current is written as follows:

21 $$\left\{ {\begin{array}{*{20}c} {\frac{{d\hat{i}_{\alpha } }}{dt} = - \frac{R}{{L_{q} }}\hat{i}_{\alpha } + \frac{1}{{L_{q} }}E_{\alpha } + \frac{1}{{L_{q} }}k_{1} \sqrt {\left| {\tilde{i}_{\alpha } } \right|} \cdot sign(\tilde{i}_{\alpha } ) + \frac{1}{{L_{q} }}\int {k_{2} sign(\tilde{i}_{\alpha } )dt} } \\ {\frac{{d\hat{i}_{\beta } }}{dt} = - \frac{R}{{L_{q} }}\hat{i}_{\beta } + \frac{1}{{L_{q} }}E_{\beta } + \frac{1}{{L_{q} }}k_{1} \sqrt {\left| {\tilde{i}_{\beta } } \right|} \cdot sign(\tilde{i}_{\beta } ) + \frac{1}{{L_{q} }}\int {k_{2} sign(\tilde{i}_{\beta } )dt} } \\ \end{array} } \right.$$

When the state variable $\tilde{i}_{\alpha } ,\tilde{i}_{\beta }$ reaches the sliding surface, it remains stable on the surface, indicating that the estimate is close to the actual value (i.e., $\tilde{i}_{\alpha } = 0,\tilde{i}_{\beta } = 0$). Based on the principle of equivalent control, the estimated back-EMF can be expressed as follows:

22 $$\left\{ {\begin{array}{*{20}c} {\hat{E}_{\alpha } = - k_{1} \sqrt {\left| {\tilde{i}_{\alpha } } \right|} \cdot sign(\tilde{i}_{\alpha } ) - \int {k_{2} sign(\tilde{i}_{\alpha } )dt} } \\ {\hat{E}_{\beta } = - k_{1} \sqrt {\left| {\tilde{i}_{\beta } } \right|} \cdot sign(\tilde{i}_{\beta } ) - \int {k_{2} sign(\tilde{i}_{\beta } )dt} } \\ \end{array} } \right.$$

In Eq. (22), the linear term $- k_{1} \sqrt {\left| {\tilde{i}_{\alpha } } \right|} \cdot sign(\tilde{i}_{\alpha } )$ determines the response speed of the STSMO, while the integral term $- \int {k_{2} sign(\tilde{i}_{\alpha } )dt}$ directly affects the system’s chattering level. Since these coefficients jointly determine the estimation performance of the back-EMF, their appropriate selection plays a crucial role in the observation efficiency of the STSMO.

## Improved STSMO method

### Design of a variable-gain linear super-twisting sliding mode observer (VGLSTSMO)

Reference 22 proposes a VGLSTSMO that effectively improves the system robustness of the traditional STSMO, which is limited by fixed gains. This improvement is achieved by introducing a linear compensation term for observation errors and an adaptive adjustment mechanism for the sliding mode coefficients.

By incorporating a linear correction term into the traditional super-twisting algorithm, a Linear super-twisting SMO (LSTSMO) is designed, and its mathematical model can be expressed as follows:

23 $$\left\{ {\begin{array}{*{20}c} {\frac{{d\hat{x}_{1} }}{dt} = - k_{1} \sqrt {\left| {x_{1} - \hat{x}_{1} } \right|} \cdot sign(x_{1} - \hat{x}_{1} ) - k_{3} (x_{1} - \hat{x}_{1} ) + \hat{x}_{2} + \rho_{1} } \\ {\frac{{d\hat{x}_{2} }}{dt} = - k_{2} \cdot sign(x_{1} - \hat{x}_{1} ) - k_{4} (x_{1} - \hat{x}_{1} ) + \rho_{2} } \\ \end{array} } \right.$$

where ${k}_{i}$ is the designed gain parameter.

Reference^23^ provides a rigorous proof of the convergence of this method based on Lyapunov stability theory, thereby establishing a solid theoretical foundation for the stability and reliability of the observer.

Assume that the disturbance term is globally bounded as:

24 $$\begin{gathered} \left\{ {\begin{array}{*{20}c} { - \delta_{1} \sqrt {\left| {\hat{x}_{1} } \right|} - \delta_{4} \left| {\hat{x}_{1} } \right| \le \rho_{1} \le \delta_{1} \sqrt {\left| {\hat{x}_{1} } \right|} + \delta_{4} \left| {\hat{x}_{1} } \right|} \\ { - \delta_{2} - \delta_{3} \left| {\hat{x}_{1} } \right| \le \rho_{2} \le \delta_{2} + \delta_{3} \left| {\hat{x}_{1} } \right|} \\ \end{array} } \right. \hfill \\ \delta_{i} \ge 0 \hfill \\ \end{gathered}$$

The estimated current value in the αβ-axis is selected as the state variable and substituted into Eq. (23). Based on this, the current estimation equation derived from the VGLSTSMO can be expressed as follows:

25 $$\left\{ {\begin{array}{*{20}c} {\frac{{d\hat{i}_{\alpha } }}{dt} = - \frac{1}{{L_{q} }}k_{1} \sqrt {\left| {\tilde{i}_{\alpha } } \right|} \cdot sign(\tilde{i}_{\alpha } ) - \frac{1}{{L_{q} }}k_{3} \tilde{i} - \frac{1}{{L_{q} }}\int {k_{2} sign(\tilde{i}_{\alpha } )dt} - \frac{1}{{L_{q} }}\int {k_{4} \tilde{i}_{\alpha } } dt + \rho } \\ {\frac{{d\hat{i}_{\beta } }}{dt} = - \frac{1}{{L_{q} }}k_{1} \sqrt {\left| {\tilde{i}_{\beta } } \right|} \cdot sign(\tilde{i}_{\beta } ) - \frac{1}{{L_{q} }}\int {k_{2} sign(\tilde{i}_{\beta } )dt} - \frac{1}{{L_{q} }}k_{3} \tilde{i}_{\beta } - \frac{1}{{L_{q} }}\int {k_{4} \tilde{i}_{\beta } } dt + \rho } \\ \end{array} } \right.$$

where $\hat{i}_{\alpha } ,\hat{i}_{\beta }$ is the estimated stator current and $\tilde{i} = \hat{i} - i$ is the current estimation error.

After removing the disturbance term, the current error can be expressed as:

26 $$\left\{ {\begin{array}{*{20}c} {\frac{{d\hat{i}_{\alpha } }}{dt} = - \frac{R}{{L_{q} }}\tilde{i}_{\alpha } + \frac{1}{{L_{q} }}E_{\alpha } - \frac{1}{{L_{q} }}k_{1} \sqrt {\left| {\tilde{i}_{\alpha } } \right|} \cdot sign(\tilde{i}_{\alpha } ) - \frac{1}{{L_{q} }}\int {k_{2} sign(\tilde{i}_{\alpha } )dt} - \frac{1}{{L_{q} }}k_{3} \tilde{i}_{\alpha } - \frac{1}{{L_{q} }}k_{4} \tilde{i}_{\alpha } dt} \\ {\frac{{d\hat{i}_{\beta } }}{dt} = - \frac{R}{{L_{q} }}\tilde{i}_{\beta } + \frac{1}{{L_{q} }}E_{\beta } - \frac{1}{{L_{q} }}k_{1} \sqrt {\left| {\tilde{i}_{\beta } } \right|} \cdot sign(\tilde{i}_{\beta } ) - \frac{1}{{L_{q} }}\int {k_{2} sign(\tilde{i}_{\beta } )dt} - \frac{1}{{L_{q} }}k_{3} \tilde{i}_{\beta } - \frac{1}{{L_{q} }}k_{4} \tilde{i}_{\beta } dt} \\ \end{array} } \right.$$

When the system trajectory reaches and remains on the sliding surface, the estimated back-EMF can be expressed as:

27 $$\left\{ {\begin{array}{*{20}c} {\hat{E}_{\alpha } = - L_{q} k_{1} \left| {\tilde{i}_{\alpha } } \right| \cdot sign(\tilde{i}_{\alpha } ) - L_{q} k_{2} \tilde{i}_{\alpha } - L_{q} \int {k_{2} sign(\tilde{i}_{\alpha } )dt} - k_{3} \tilde{i}_{\alpha } } \\ {\hat{E}_{\beta } = - L_{q} k_{1} \left| {\tilde{i}_{\beta } } \right| \cdot sign(\tilde{i}_{\beta } ) - L_{q} k_{2} \tilde{i}_{\beta } - L_{q} \int {k_{2} sign(\tilde{i}_{\beta } )dt} - k_{3} \tilde{i}_{\beta } } \\ \end{array} } \right.$$

**Fig. 2:**
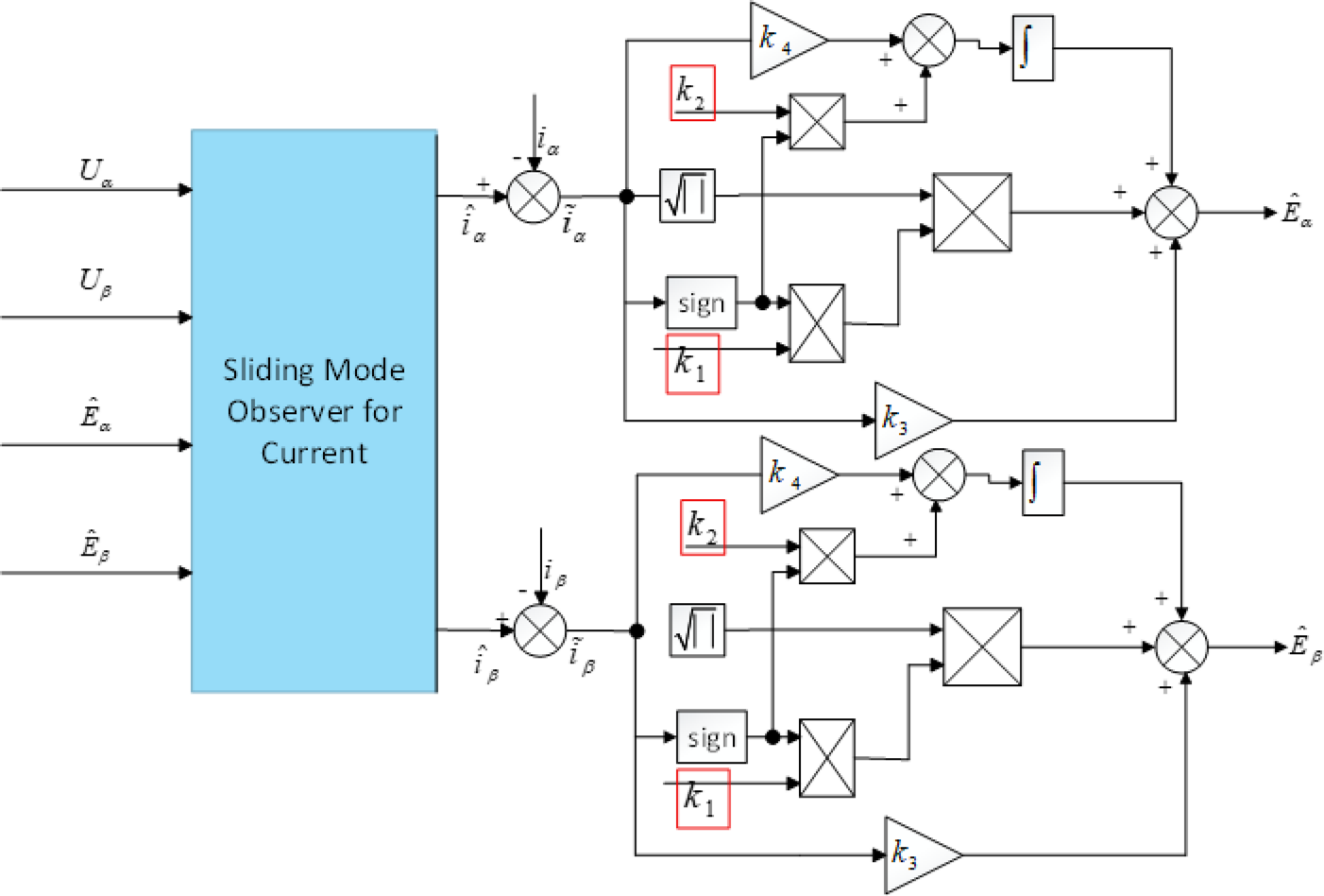
Block Diagram of the VGLSTSMO Implementation Principle.

Following the design procedure described above, Fig. 2 presents the block diagram illustrates the implementation principle of the VGLSTSMO.

Slip gain adjustment is incorporated into the back-EMF design, where the slip coefficients ${k}_{1}$ and ${k}_{2}$ vary in real time with the motor speed ${N}_{est}$. Their gain parameters are defined as follows:

28 $$k_{1} = \eta_{1} f(N_{est} ),k_{2} = \eta_{2} f(N_{est}^{2} )$$

29 $$f(N_{est} ) = (2c - 1)N_{est} + c,\frac{1}{2} \le c \le 1$$

where ${\eta }_{1}$ , ${\eta }_{2}$ are adjustable coefficients, c is a constant, and $f({N}_{est})$ is a function of motor speed.

**Fig. 3:**
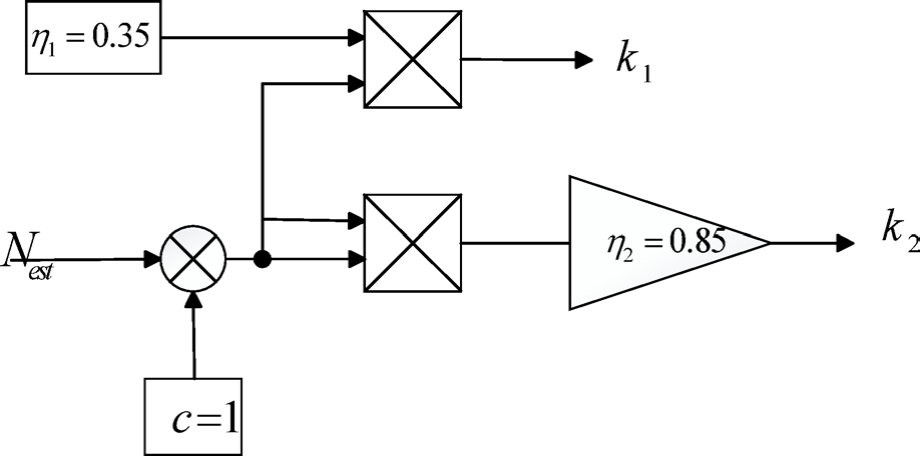
Schematic Diagram Illustrating the Implementation of the slip coefficients ${k}_{1}$ and ${k}_{2}$.

The implementation of the slip gain adjustment based on Eq. (28) and Eq. (29) is shown in Fig. 3, where the coefficients ${k}_{1}$ and ${k}_{2}$ are generated in real time as functions of the estimated motor speed ${N}_{est}$.

**Fig. 4:**
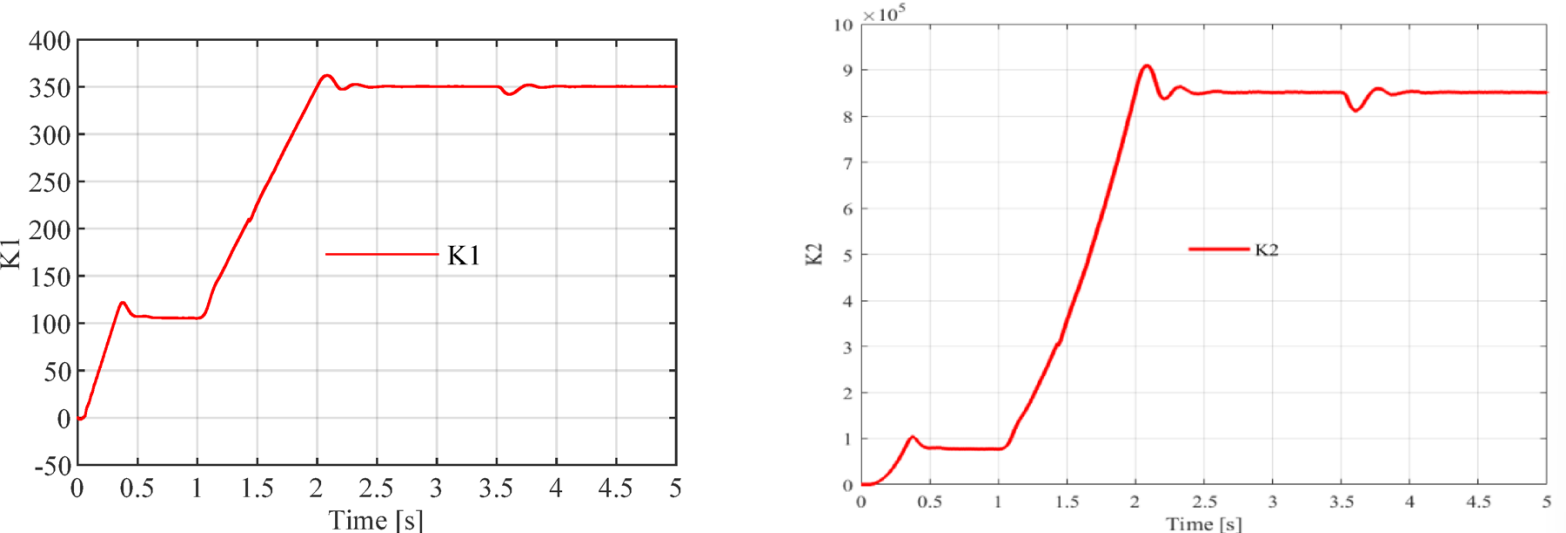
Dynamic responses of the sliding coefficients ${k}_{1}$ and ${k}_{2}$ in the sliding mode current observer.

To verify the performance of the sliding mode current observer, the dynamic behaviors of the sliding coefficients ${k}_{1}$ and ${k}_{2}$ are illustrated in Fig. 4.

From Eq. (24), it follows that when the designed observer is stable, there exists a constant $\uplambda$ that satisfies the following equation:

30 $$\left\{ {\begin{array}{*{20}c} {\delta_{i} = \lambda_{i} N_{est} } & {i = 1,2} \\ {\delta_{j} = \lambda_{j} N_{est}^{2} } & {j = 3,4} \\ \end{array} } \right.$$

It follows that the aforementioned global boundedness condition can be expressed as:

31 $$\left\{ {\begin{array}{*{20}c} {|\rho_{1} | \le \delta_{1} \sqrt {\left| {\tilde{i}_{\alpha \beta } } \right|} + \delta_{2} \left| {\tilde{i}_{\alpha \beta } } \right| = \lambda_{1} N_{est} \sqrt {\left| {\tilde{i}_{\alpha \beta } } \right|} + \lambda_{2} N_{est} \left| {\tilde{i}_{\alpha \beta } } \right|} \\ {|\rho_{2} | \le \delta_{3} + \delta_{4} \left| {\tilde{i}_{\alpha \beta } } \right| = \lambda_{3} N_{est}^{2} + \lambda_{4} N_{est}^{2} \left| {\tilde{i}_{\alpha \beta } } \right|} \\ \end{array} } \right.$$

After satisfying the stability condition, appropriate parameters are selected to guarantee that the system meets the stability requirements.

32 $$k_{1} = \eta_{1} f(N_{est} ) > 2\max (\lambda_{1} N_{est} ,\sqrt {\lambda_{3} } N_{est} )$$

33 $$\left\{ {\begin{array}{*{20}c} \begin{gathered} k_{2} = \eta_{2} f(N_{est}^{2} ) > \max (\eta_{1} \frac{{\lambda_{1} \eta_{1} + \frac{1}{8}\lambda_{1}^{2} + \lambda_{3} }}{{2(\frac{1}{2}\eta_{1} - \lambda_{1} )}}N_{est}^{2} ,(\frac{{(\eta_{3} \lambda_{1} + \frac{1}{2}\eta_{1} \lambda_{2} )^{2} }}{{2\eta_{3} (\eta_{3} - 2\lambda_{2} )}} \hfill \\ + \frac{{(\lambda_{3} + \frac{3}{2}\eta_{1} \lambda_{1} )\eta_{3} - 2(\eta_{3} - \frac{1}{4}\lambda_{2} )\eta_{1}^{2} }}{{\eta_{3} - 2\lambda_{2} }})N_{est}^{2} ) \hfill \\ \end{gathered} \\ {k_{3} = \eta_{2} f(N_{est} ) = 2\delta_{3} + \frac{{\sqrt {2\delta_{4} } }}{2} > \max (\frac{3}{8}\delta_{3} + \frac{1}{4}\sqrt {\frac{9}{4}\delta_{3}^{2} + 8\delta_{4} } ,2\delta_{3} )} \\ \end{array} } \right.$$

when the design parameters ${\upeta }_{1},{\upeta }_{2}$, ${\lambda }_{1}$ and ${\lambda }_{2}$ have been selected, the following expressions for the constants ${c}_{1}$ and ${c}_{2}$ are given:

34 $$\left\{ {\begin{array}{*{20}c} {c_{1} = \eta_{1} \frac{{\lambda_{1} \eta_{1} + \frac{1}{8}\lambda_{1}^{2} + \lambda_{3} }}{{2(\frac{1}{2}\eta_{1} - \lambda_{1} )}}} \\ {c_{2} = \frac{{(\eta_{3} \lambda_{1} + \frac{1}{2}\eta_{1} \lambda_{2} )^{2} }}{{2\eta_{3} (\eta_{3} - 2\lambda_{2} )}} + \frac{{(\lambda_{3} + \frac{3}{2}\eta_{1} \lambda_{1} )\eta_{3} - 2(\eta_{3} - \frac{1}{4}\lambda_{2} )\eta_{1}^{2} }}{{\eta_{3} - 2\lambda_{2} }}} \\ \end{array} } \right.$$

Stability analysis of the proposed method, as demonstrated in Reference 22, shows that the parameters can be adaptively adjusted according to the actual motor speed. This ensures global system stability while simultaneously preventing oscillations in the medium–high speed range of the motor.

### Principle of adaptive back-EMF fusion

Adaptive adjustment is applied to the estimated back-EMF in the VGLSTSMO design, which can be expressed as:

35 $$\left\{ {\begin{array}{*{20}c} {\frac{{d\hat{E}_{\alpha } }}{dt} = - \hat{\omega }_{e} \hat{E}_{\beta } - H\tilde{E}_{\alpha } = - \hat{\omega }_{e} \hat{E}_{\beta } - H(\hat{E}_{\alpha } - E_{\alpha } )} \\ {\frac{{d\hat{E}_{\beta } }}{dt} = \hat{\omega }_{e} \hat{E}_{\alpha } - H\tilde{E}_{\beta } = \hat{\omega }_{e} \hat{E}_{\alpha } - H(\hat{E}_{\beta } - E_{\beta } )} \\ \end{array} } \right.$$

where ${\widehat{E}}_{\alpha }$ , ${\widehat{E}}_{\beta }$ is the estimated value of the PMSMs back-EMF, $\hat{\omega }_{e}$ is the estimated rotor electrical speed, and H is the gain coefficient of the adaptive back-EMF observer. An excessively high gain slows down the response, whereas an excessively low gain causes waveform distortion.

The back-EMF estimation error equation for the PMSM can be expressed as:

36 $$\left\{ {\begin{array}{*{20}c} {\frac{{d\hat{E}_{\alpha } }}{dt} = - \tilde{\omega }_{e} \hat{E}_{\beta } - \hat{\omega }_{e} \hat{E}_{\beta } - H\tilde{E}_{\alpha } } \\ {\frac{{d\hat{E}_{\beta } }}{dt} = \tilde{\omega }_{e} \hat{E}_{\alpha } - \hat{\omega }_{e} \hat{E}_{\alpha } - H\tilde{E}_{\beta } } \\ \end{array} } \right.$$

The observer realizes the estimation process of ${\widehat{E}}_{\alpha }$ and ${\widehat{E}}_{\beta }$ based on the adaptive law, where the gain coefficient is set to $H=3000$ in this paper.

Based on Lyapunov stability theory, system stability can be demonstrated by constructing an appropriate Lyapunov function and analyzing its derivative.

The Lyapunov function V and its derivative can be expressed as:

37 $$\begin{gathered} V = \frac{1}{2}E(X)^{T} E(X) + \frac{1}{2\gamma }\tilde{\omega }_{e}^{2} = \frac{1}{2}(\tilde{e}_{\alpha }^{2} + \tilde{e}_{\beta }^{2} ) + \frac{1}{2\gamma }\tilde{\omega }_{e}^{2} \hfill \\ E(X) = \left[ {\hat{e}_{\alpha } } \right.\left. {\hat{e}_{\beta } } \right]^{T} ,\tilde{\omega }_{e} = \hat{\omega }_{e} - \omega_{e} \hfill \\ \end{gathered}$$

where $\upgamma$ is a positive constant. The value of the constant $\gamma$ used in the observer design is set to $1\times {10}^{3}$.

38 $$\begin{gathered} \dot{V} = E(X)^{T} \dot{E}(X) + \frac{1}{\gamma }\tilde{\omega }_{e} \tilde{\omega }_{e} = (\hat{e}_{\alpha } \tilde{e}_{\alpha } + \hat{e}_{\beta } \tilde{e}_{\beta } ) + \frac{1}{\gamma }\hat{\omega }_{e} \tilde{\omega }_{e} \hfill \\ = - H(\hat{e}_{\alpha }^{2} + \hat{e}_{\beta }^{2} + \hat{\omega }_{e} (\hat{e}_{\alpha } \hat{e}_{\beta } - \hat{e}_{\beta } \hat{e}_{\alpha } + \frac{1}{\gamma }\hat{\omega }_{e} ) \hfill \\ \end{gathered}$$

The Eq. (37) satisfies the Lyapunov stability condition, i.e., V is positive definite. When $\dot{V} < 0$ , the designed observer is stable, and the adaptive design law can be expressed as follows:

39 $$\left\{ {\begin{array}{*{20}c} {H > 0} \\ {\hat{\omega }_{e} = \gamma \int {(\hat{E}_{\beta } \tilde{E}_{\alpha } - \hat{E}_{\alpha } \tilde{E}_{\beta } )dt} } \\ \end{array} } \right.$$

From the observed back-EMF signal, the rotor’s rotational speed and position information are extracted using a PLL. This method incorporates a PI controller to suppress high-frequency components in the system without the need for a low-pass filter design. It also enables more accurate acquisition of rotor position and speed, thereby enhancing the estimation precision.

## Smooth switching strategy for full-speed-range sensorless composite control

### Linear weighted switching strategy

In order to achieve a smooth transition between control strategies in the low-speed and medium–high-speed regions, a smoothing switching mechanism is designed. Two speed thresholds ${\upomega }_{1}$ and ${\upomega }_{2}$ are set within the rated speed range of the motor, dividing the operating domain into three regions: the low-speed domain (${\upomega <\upomega }_{1}$), the medium–high-speed domain (${\upomega >\upomega }_{2}$), and the transition domain (${{\upomega }_{1}<\upomega <\upomega }_{2}$). When the motor in the transition domain, the final estimated rotor position is obtained through a weighted fusion of the results from the two observers.

**Fig. 5:**
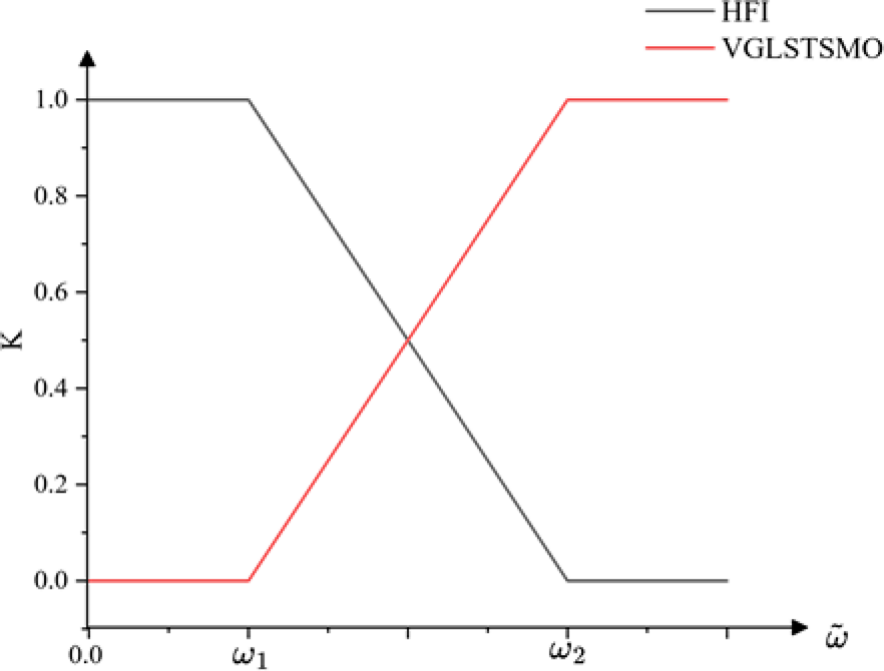
Diagram of the Linear Weighted Switching Principle.

The schematic diagram of the linear weighting switching principle is shown in Fig. 5.

The weighting coefficient k can be expressed as:

40 $$k(\tilde{\omega }) = \left\{ {\begin{array}{*{20}c} 0 & {\tilde{\omega } < \omega_{1} } \\ {\frac{{\tilde{\omega } - \omega_{1} }}{{\omega_{2} - \omega_{1} }}} & {\omega_{1} \le \tilde{\omega } \le \omega_{2} } \\ 1 & {\tilde{\omega } > \omega_{2} } \\ \end{array} } \right.$$

The estimation equation for the weighted switching control strategy is given as:

41 $$\left\{ {\begin{array}{*{20}c} {\tilde{\omega } = k\tilde{\omega }_{SMO} + (1 - k)\tilde{\omega }_{HFI} } \\ {\tilde{\theta } = k\tilde{\theta }_{SMO} + (1 - k)\tilde{\theta }_{HFI} } \\ \end{array} } \right.$$

where $\tilde{\omega }_{HFI} ,\tilde{\theta }_{HFI}$ are the estimated rotor position and speed from the low-speed strategy, and $\tilde{\omega }_{SMO} ,\tilde{\theta }_{SMO}$ are the corresponding estimates from the medium–high-speed strategy.

By linearly blending the outputs of the two methods in the transition region, a smooth transition in the motor control system is intended. However, it may induce position and speed fluctuations.

### Improved switching strategy

The HFI with square-wave method is employed for low speeds, while the VGLSTSMO is employed in the medium- high-speed region in this paper. Due to the use of different estimation strategies to make the motor smoothly transit from the low-speed range to the medium–high-speed region. Therefore, a sine-weighted switching function is designed in this paper to achieve smooth switching in the transition region.

The expression for the improved weighting function K is given by:

42 $$\begin{gathered} k(\tilde{\omega }) = \left\{ {\begin{array}{*{20}c} {0,} \\ {\frac{1}{2}\sin [\lambda (\tilde{\omega } - \hat{\omega }_{0} )] + \frac{1}{2},} \\ 1 \\ \end{array} } \right.\begin{array}{*{20}c} {\tilde{\omega } < \omega_{1} } \\ {\omega_{1} \le \tilde{\omega } \le \omega_{2} } \\ {\tilde{\omega } > \omega_{2} } \\ \end{array} \hfill \\ \lambda = \frac{\pi }{{\omega_{2} - \omega_{1} }},\hat{\omega }_{0} = \frac{{\omega_{1} + \omega_{2} }}{2} \hfill \\ \end{gathered}$$

**Fig. 6:**
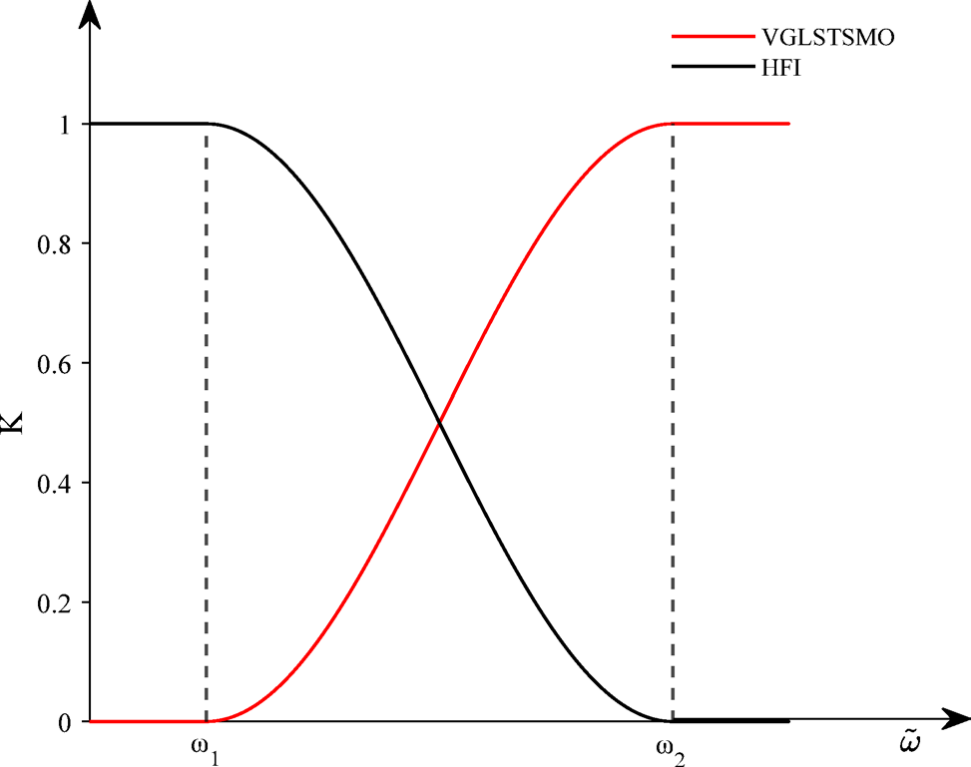
Diagram of the Sine-weighted Function Switching Principle.

Substituting Eq. (42) into Eq. (41) yields the corresponding weight distribution for the two methods. In this way, smooth switching can be achieved. The schematic diagram illustrating this principle is shown in Fig. 6.

## Simulation verification and results analysis

In order to verify the effectiveness of the proposed VGLSTSMO in enhancing system robustness and the optimization effect of the sine-weighted switching function strategy on full-speed-range control, the FOC control strategy is adopted, with the value set to 0. Simulation models are constructed in Simulink, and comparative verification is conducted. The parameters of the PMSM and control settings used in the simulation are listed in Table 2.

**Table 2:** Parameters of PMSM and Control Settings Used in the Simulation.

Parameters	Value
Stator Resistance[Ω]	0.958
Number of Pole Pairs	4
d-axis Inductance $[mH]$	5.25
q-axis Inductance [$mH]$	12
Rotational Inertia $[\mathrm{kg}\bullet {\mathrm{m}}^{2}]$	0.03
Flux Linkage $[Wb]$	0.1827
STSMO Parameters	${k}_{1}=450,{k}_{2}=250$
VGLSTSMO Parameters	${k}_{3}=100,{k}_{4}=50$
PLL-HFI Parameters	${k}_{P}=86.6,{k}_{i}=5000$
PLL-STSMO and VGLSTSMO	${k}_{p}=80,{k}_{2}=1600$

### Sensorless control based on the proposed VGLSTSMO method

**Fig. 7:**
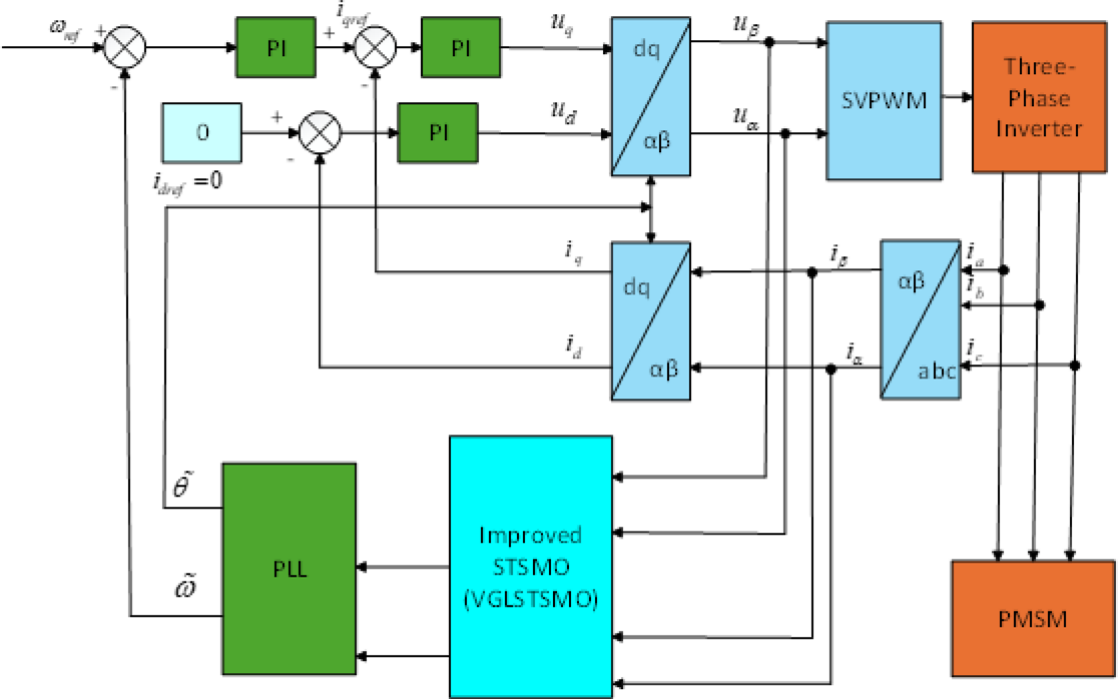
VGLSTSMO sensorless control model diagram.

The VGLSTSMO sensorless control model is depicted in Fig. 7, with related comparative simulations and analyses presented in detail. To evaluate the performance of the VGLSTSMO, a simulation comparison between the conventional STSMO and the VGLSTSMO is conducted within the medium-to-high-speed range. The reference speed is set at 1000 rpm, with a simulation duration of 2 s. A load torque of 2 N·m is applied at 0.8 s.

**Fig. 8:**
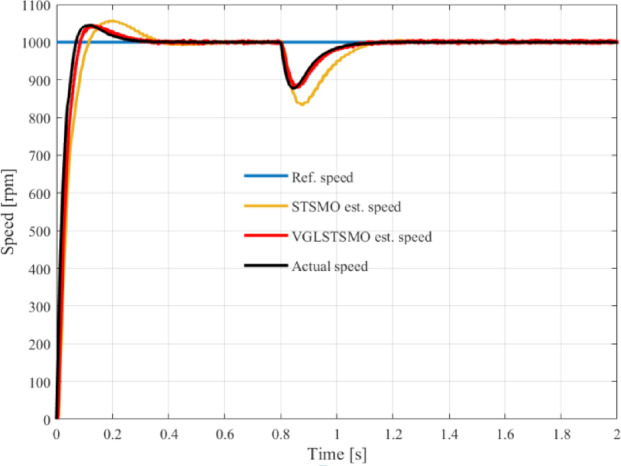
Comparison of STSMO and VGLSTSMO: Given Speed, Estimated Speed, and Actual PMSM Model Speed.

**Fig. 9:**
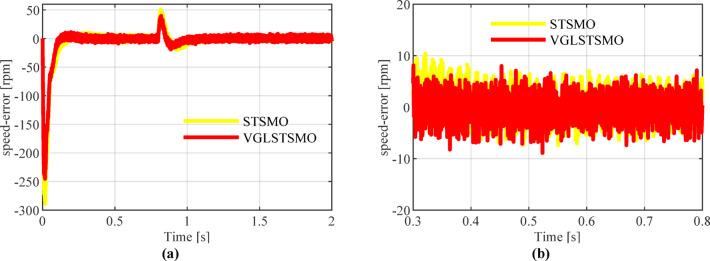
Comparison of Speed Error between STSMO and VGLSTSMO: (**a**) Overall view; (**b**) Enlarged view.

**Fig. 10:**
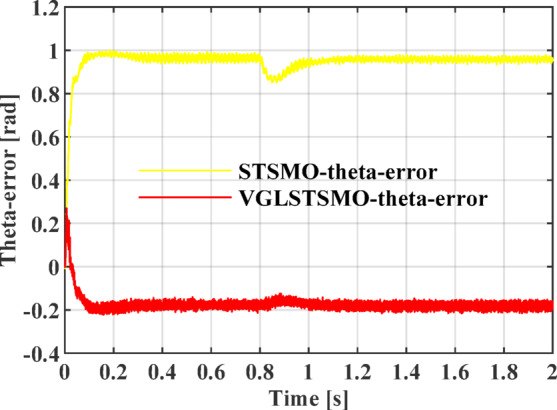
Comparison of Rotor Position Angle Error between STSMO and VGLSTSMO.

As shown in Figs. 8, 9 and 10, the STSMO estimates a maximum overshoot in the estimated rotational speed at 0.203 s, with an overshoot magnitude of 5.9%. After load application, it reaches basic stability after 0.14 s, and the rotor position error stabilizes around 0.9 rad. The VGLSTSMO, on the other hand, reaches its maximum overshoot at 0.129 s with a 4.8% overshoot, which is lower than that of the STSMO, thus reducing oscillations to some extent. After load application, it achieves basic stability within 0.08 s, demonstrating faster recovery. The rotor position error stabilizes around 0.2 rad. In contrast, the VGLSTSMO exhibits superior performance in the medium–high-speed range, with enhanced stabilization effectiveness.

### Full-speed range sensorless composite control

**Fig. 11:**
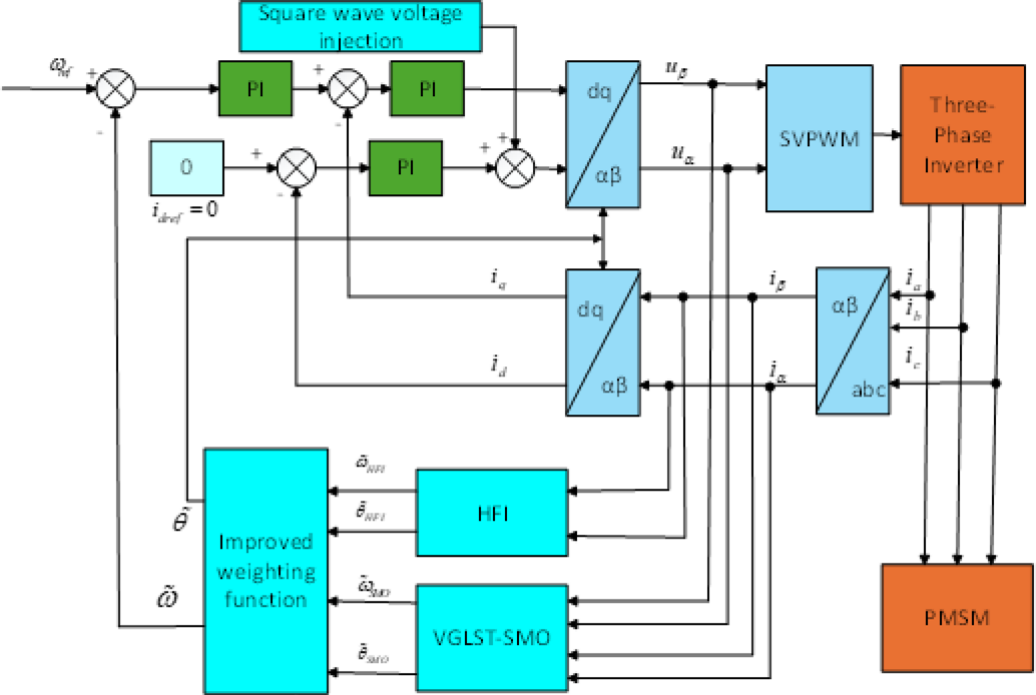
Full-Speed Range Sensorless Control Block Diagram.

Figure 11 illustrates the block diagram of the full-speed-range sensorless control. A comprehensive comparison is conducted between the proposed sine-weighted switching method and the traditional linear weighting method. The reference speed is applied using a ramp function. The maximum speed is set to 1000 rpm, with a simulation duration of 5 s. A load torque of 2 N·m is applied at 3.5 s. The full-speed range is selected with lower and upper limits of 300 rpm and 600 rpm, respectively. For speeds below 300 rpm, the proposed high-frequency square-wave voltage injection method is employed to estimate the rotor position. For speeds above 600 rpm, the VGLSTSMO is used for rotor position estimation. In the intermediate speed range between 300 and 600 rpm, the rotor position is estimated using both the HFI method and the VGLSTSMO.

**Fig. 12:**
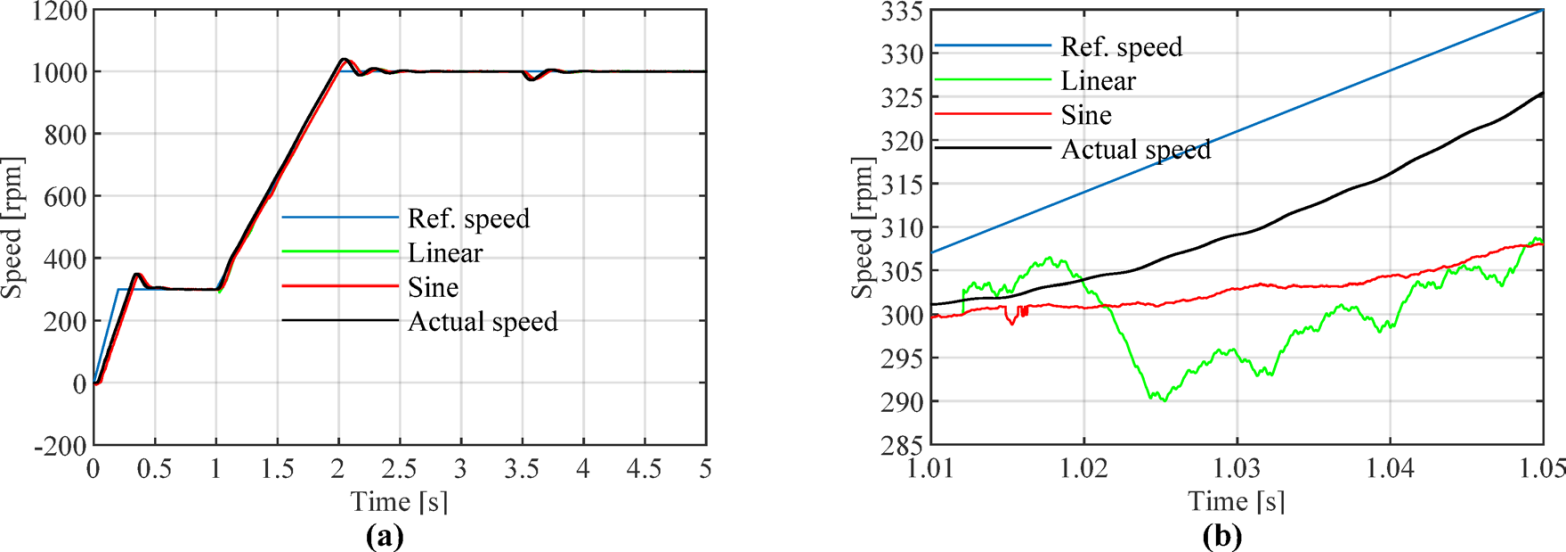
Comparison of reference, estimated, and actual rotational speeds for linear and sine weighting functions: (**a**) Overall view; (**b**) Enlarged view at switching onset.

As shown in Fig. 12, during the initial stage of the speed transition (approximately 1.012 s), the linear weighting method exhibits minor oscillations. Around 1.017 s, a significant speed jump occurs, causing the estimated speed to abruptly drop to 290 rpm.

**Fig. 13:**
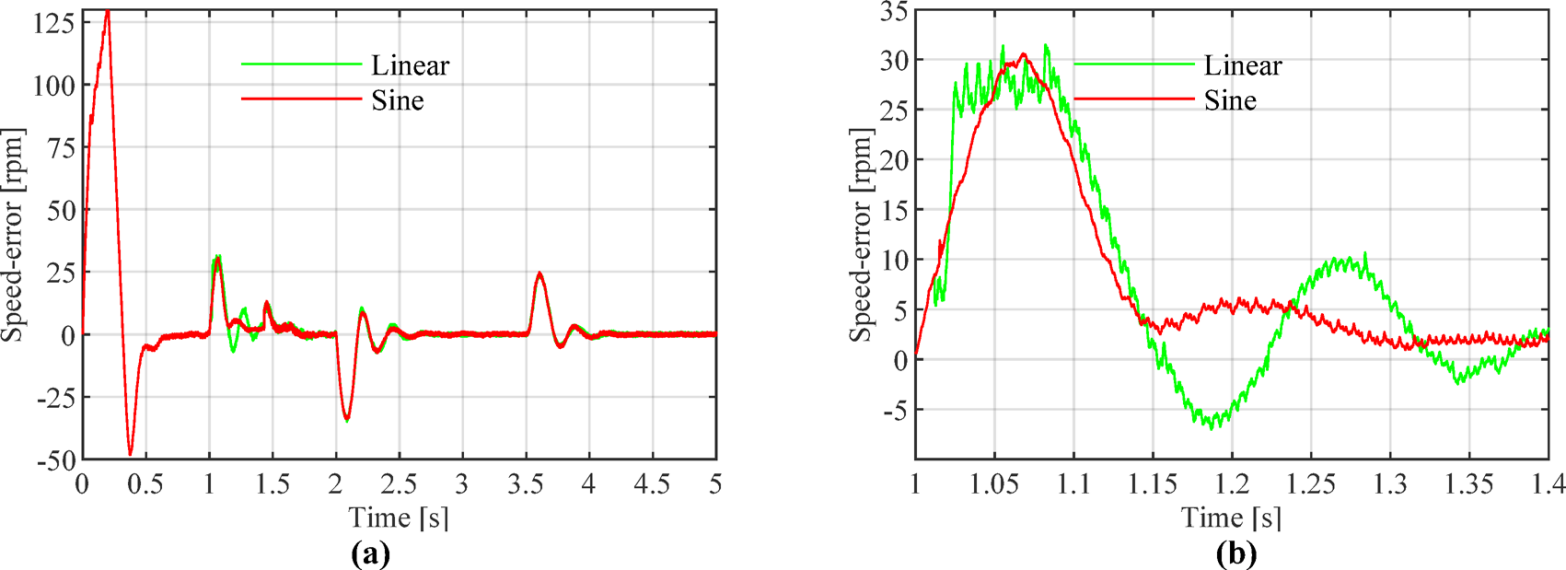
Comparison of Rotational Speed Errors between Sine and Linear Weighting Functions: (a) Overall view; (**b**) Enlarged view in the switching-speed range.

**Fig. 14:**
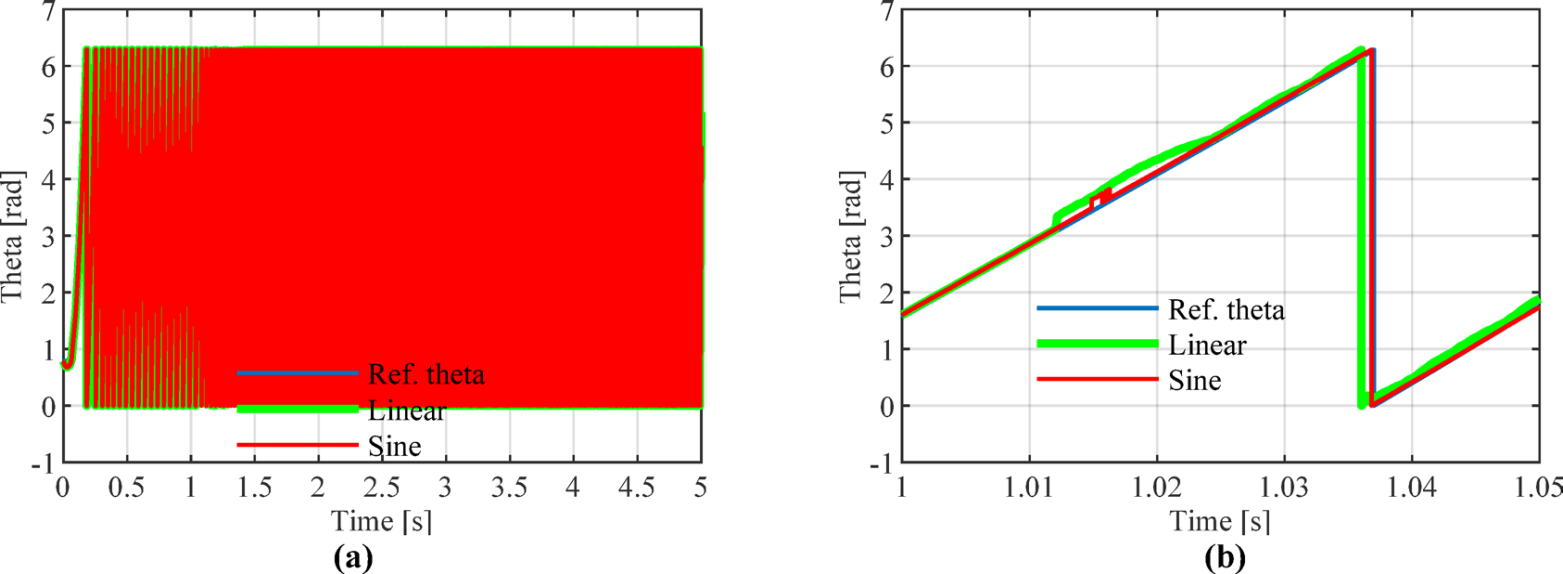
Comparison of Rotor Position Angle Estimation between Linear and Sine Weighting Functions: (**a**) Overall view; (**b**) Enlarged view at switch initiation.

**Fig. 15:**
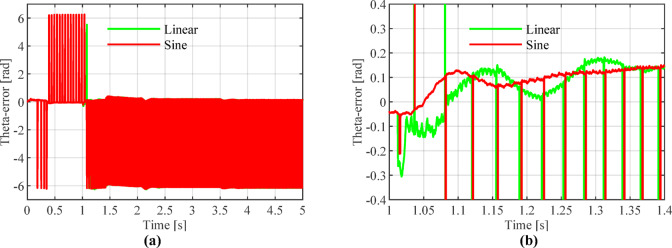
Comparison of Angular Errors between Linear and Sine Weighting Methods: (**a**) Overall view; (**b**) Enlarged view at switch initiation.

Figures 13, 14 and 15 show that the linear weighting method exhibits a maximum rotational speed error of 31.47 rpm throughout the transition process, gradually converging after 0.3 s. The rotor position angle error is -0.30 rad, with a transient offset time of approximately 0.012 s and a steady-state phase lag of about 0.001 rad. In contrast, the sine-weighted switching function method exhibits a maximum rotational speed error of 30.4 rpm, stabilizing rapidly within 0.15 s. Its rotor position angle error is -0.21 rad, with a transient offset time of only 0.0014 s and a steady-state phase lag reduced to 0.0002 rad, indicating higher estimation accuracy. Regarding waveform characteristics, the linear weighting method exhibits noticeable fluctuations throughout the entire transition interval. In contrast, the sine-weighted switching function method demonstrates superior stability and smoothness, with no significant chattering, thereby exhibiting better transient performance and robustness. The results indicate that the proposed sine-weighted switching function strategy effectively enhances the smoothness and overall stability of the system switching process.

This paper presents a comparative simulation analysis of the performance of the STSMO and the VGLSTSMO within a full-speed-range control system that employs a sine-weighted switching strategy. The simulation data used are consistent with the previously mentioned full-speed-range specifications.

**Fig. 16:**
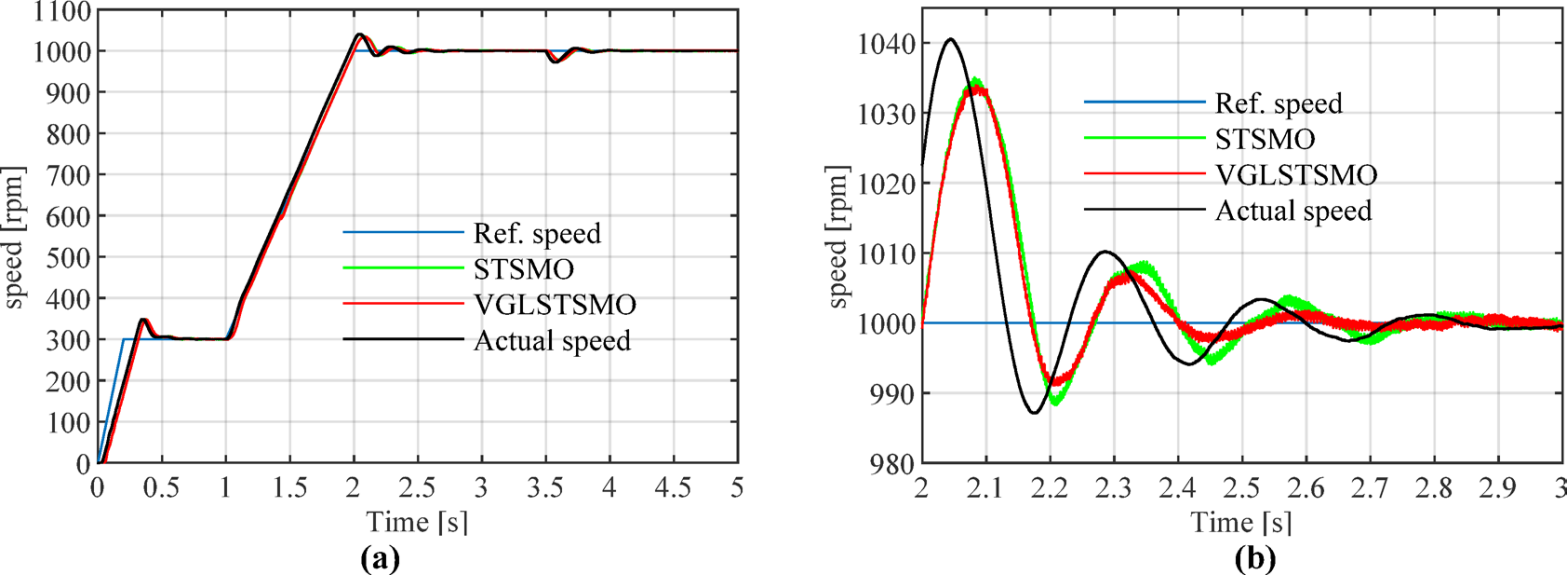
Comparison of Estimated Rotational Speeds between STSMO and VGLSTSMO under a Sinusoidal Switching Function: (**a**) Overall view; (**b**) Enlarged view of local speed after.

**Fig. 17:**
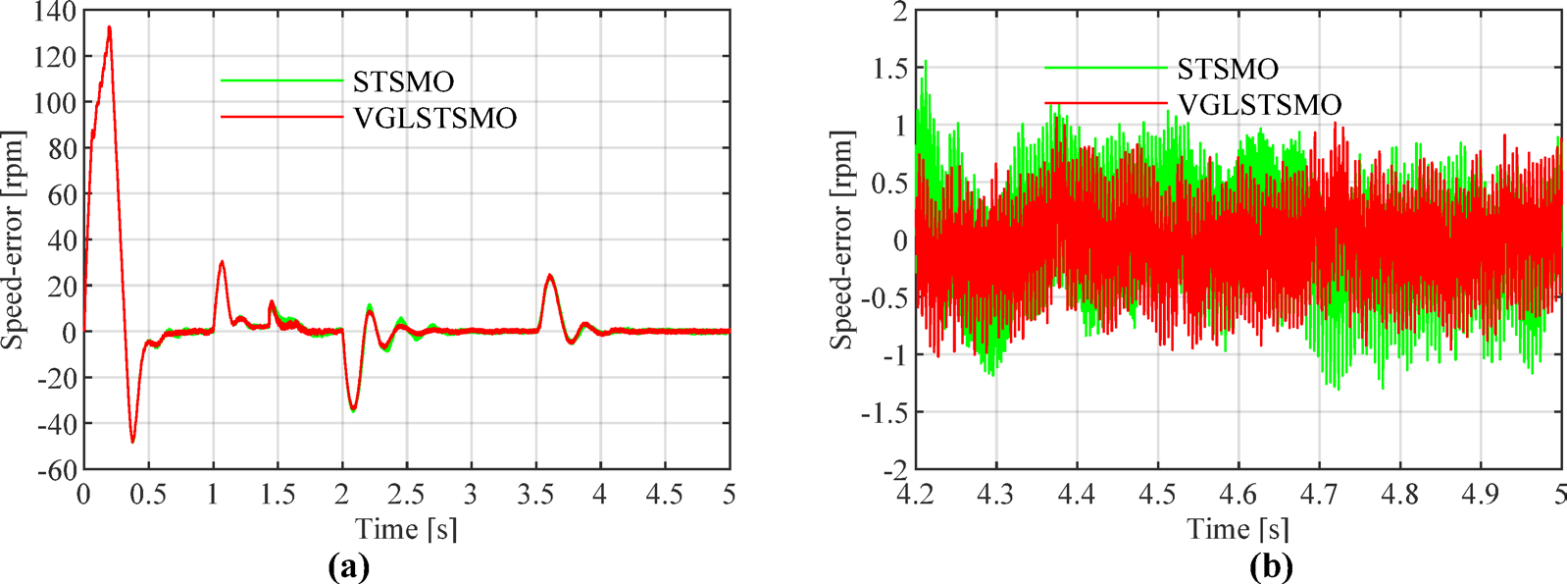
Comparison of Speed Errors between STSMO and VGLSTSMO under a Sinusoidal Switching Function: (**a**) Overall view; (**b**) Enlarged view after basic stabilization.

**Fig. 18:**
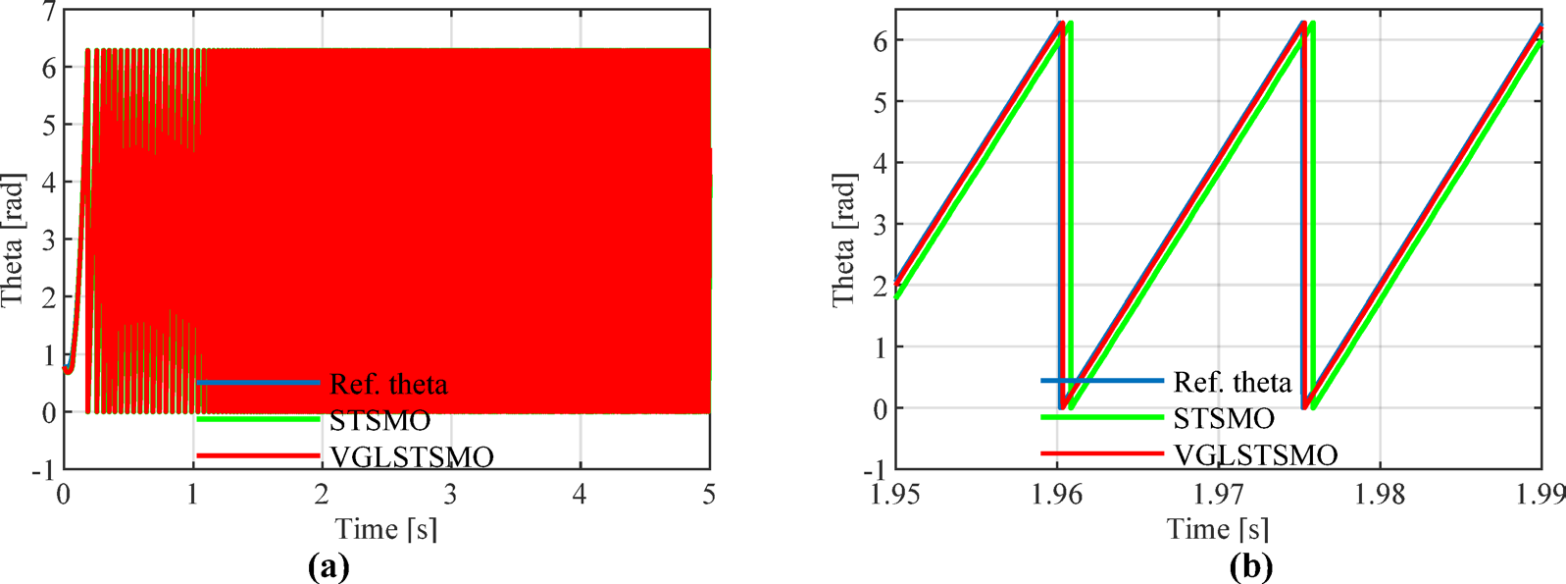
Comparison of Rotor Position Angle Estimation between STSMO and VGLSTSMO under a Sinusoidal Switching Function: (**a**) Overall view; (**b**) Enlarged view after basic stabilization.

**Fig. 19:**
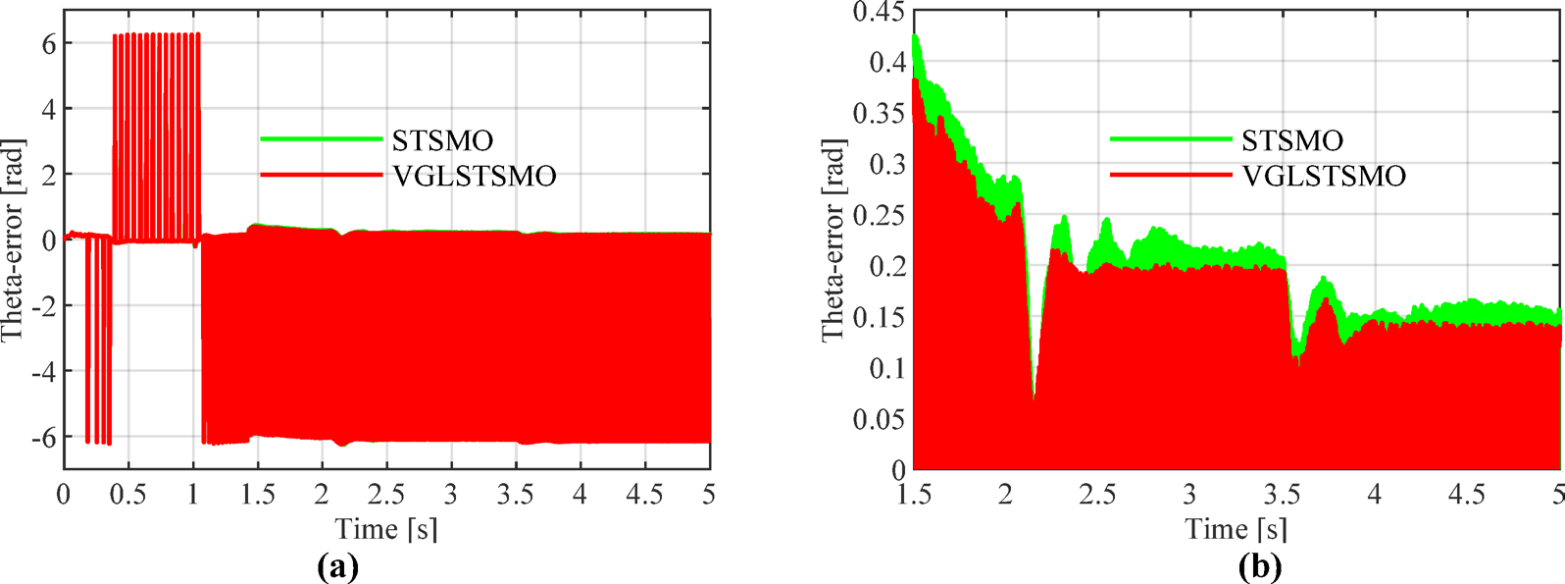
Comparison of Rotor Position Angle Errors between STSMO and VGLSTSMO under Sinusoidal Switching Functions: (**a**) Overall view; (**b**) Enlarged view.

Figures 16, 17, 18 and 19 show that after 2 s, the maximum overshoot of the estimated rotational speed for STSMO reached 3.5% and stabilized after 2.95 s. Meanwhile, the maximum overshoot for VGLSTSMO was 3.3%, and stabilized after 2.75 s. After applying a load disturbance at 3.5 s, neither the actual nor estimated rotational speeds exhibited significant fluctuations under either strategy, indicating robust disturbance rejection capability (Tables 3, 4 and 5).

**Table 3:** Performance Comparison of Different Methods in the Medium–High-Speed Range.

Mid-High-Speed Range	Maximum Overshoot [%]	Peak Time [s]	Rotor Position Estimation Error [rad]	Recovery Time from Load Disturbance [s]
STSMO	5.9	0.203	0.9	0.14
VGLSTSMO	4.8	0.129	0.2	0.08

**Table 4:** Performance Comparison of Different Switching Methods across the Full-Speed Rang.

Full Speed Rang	Maximum Speed Estimation Error [rpm]	Error Convergence Time [s]	Transition Smoothness	Switching Transient Position Error [rad]	Transient Offset Duration [s]	Steady-State Phase Lag [rad]
Linear Weighting Method	31.47	0.3	with fluctuations	0.30	0.012	0.001
Sine-type Function Weighting Method	30.4	0.15	Overall smoothness	0.21	0.0014	0.0002

**Table 5:** Performance Comparison of Different Medium-to-High-Speed Methods across the Entire Speed Range.

Full Speed Rang	Maximum Overshoot [%]	Stabilization time [s]	Speed Estimation Error Range[rpm]	Average Position Estimation Error [rad]	Steady-State Position Lag [rad]
Sine + STSMO	3.5	0.95	± 1.5	0.15	0.00065
Sine + VGLSTSMO	3.3	0.75	± 1.0	0.13	0.00013

As shown in Fig. 17, during steady-state operation, the rotational speed error fluctuation range for STSMO is ± 1.5 rpm, while VGLSTSMO further reduces this to ± 1 rpm, demonstrating superior steady-state performance and smoothness.

As shown in Fig. 18, STSMO exhibits a phase lag of approximately 0.065%, while VGLSTSMO significantly improves this to 0.013%, demonstrating superior tracking accuracy and dynamic performance.

As shown in Fig. 19, VGLSTSMO exhibits smaller estimation errors across the entire position range. Its steady-state position estimation error is 0.13 rad, outperforming STSMO’s 0.15 rad, thereby validating its superior observation accuracy and steady-state performance.

## Conclusions

This paper proposes a sensorless composite control strategy for PMSMs across the full speed range, based on HFI with square waves and VGLSTSMO. To address the medium–high-speed operation requirements, the VGLSTSMO method is introduced. By incorporating an adaptive back-EMF mechanism and a dynamic gain adjustment strategy, it overcomes the robustness limitations of traditional STSMO caused by fixed gains, significantly improving observation accuracy and controlling steady-state rotor position error within 0.2 rad. For full-speed-range switching, a smooth transition mechanism based on a sine-weighted function is designed. This effectively suppresses torque and speed fluctuations during mode switching, enabling a seamless transition from the high-frequency square-wave voltage injection method to the VGLSTSMO method. MATLAB/Simulink simulation results demonstrate that the proposed control strategy effectively suppresses speed transients and chattering phenomena, ensuring stable motor operation throughout the full speed range, from low to medium–high speeds. This validates the effectiveness and reliability of the control strategy.

## Data Availability

The data that supports the findings of this study are available within the article.
